# A three-dimensional computed tomography study of the palmar ulnar corner fragment in distal radial fractures

**DOI:** 10.1177/17531934231211570

**Published:** 2023-11-16

**Authors:** James Hubbard, David Berry, Aakash Chauhan, Chris Casstevens, Alexander Y. Shin, Reid A. Abrams

**Affiliations:** 1 Division of Hand, Upper Extremity, and Microvascular Surgery, Department of Orthopaedic Surgery, University of California, San Diego (UCSD), La Jolla, CA, USA; 2 Department of Orthopedic Surgery, Mayo Clinic, Rochester, MN, USA

**Keywords:** Anatomy, distal radius fracture, lunate facet, volar ulnar corner

## Abstract

Fixing palmar ulnar corner fragments of distal radial fractures can be challenging. We described the palmar ulnar corner fragment morphology in a retrospective cohort study of 40 patients who underwent preoperative wrist computed tomography scans. Palmar ulnar corner fractures were categorized based on articular cross-sectional area, sagittal angulation relative to the radius long axis, palmar cortical length, radioulnar width and associated palmar radiocarpal subluxation. Three types emerged: type 1 fragments involved 37% (SD 10) of the radiocarpal articular surface and were extended in the sagittal plane; type 2 fragments involved 28% (SD 10) of the articular surface and had a long palmar cortex, of which 57% had palmar carpal subluxation; and type 3 fragments involved 13% (SD 2) of the articular surface, had a short palmar cortex and all had palmar carpal subluxation. Understanding palmar ulnar corner fragment morphology may guide optimal reduction and fixation strategy and prevent palmar radiocarpal subluxation, especially in type 3 fractures.

**Level of evidence** IV

## Introduction

Understanding intra-articular distal radial fractures (DRF) involving the palmar ulnar corner (PUC) is critical, because this fracture fragment contributes to two major articular surfaces (radiocarpal and distal radioulnar joints) and is the attachment of two major wrist ligaments (short radiolunate and palmar radioulnar ligaments). Malreduction of the PUC fragment may jeopardize fracture stability, result in joint incongruity and potentially lead to palmar radiocarpal subluxation (Kitay and Mudgal, 2014; Knirk and Jupiter, 1986; Ruch et al., 2010; Trumble et al., 1994).

Several DRF classifications describe a PUC fragment (Fernandez, 2001; Medoff, 2005; Meinberg et al., 2018; Melone, 1984; Missakian et al., 1992). Melone initially described the ‘volar medial fragment’ as one of the most commonly identified distal radius intra-articular fracture fragments (Melone, 1984). Medoff used the term ‘volar rim fragment’ for the PUC fragment in his description of the common articular fracture fragments (Medoff, 2005). Fernandez’s classification recognized that the PUC fragment resulted from shear, compression, avulsion and/or combined injury mechanisms with varying displacement patterns depending on the force vector (Fernandez, 2001).

DRF fixation with anterior locking plates has become well accepted but case series have described the failure of these plates to secure the PUC fragment, resulting in displacement, palmar radiocarpal subluxation, disability and secondary procedures (Beck et al., 2014; Harness et al., 2004; Kitay and Mudgal, 2014).

Despite the awareness of poor outcomes associated with PUC fragment fixation loss, a clear description of its morphology and displacement behaviour have been limited (Benis et al., 2020). The aim of the present study was to characterize the morphology of PUC fragments found in computed tomography (CT) scans from a retrospective series of patients with intra-articular DRFs, by using three-dimensional (3D) multiplanar reconstruction (MPR).

## Methods

After approval from the institutional review board, we retrospectively studied CT scans consecutively obtained by three fellowship-trained hand surgeons at a level 1 trauma centre between January 2010 and December 2017. Scans were ordered at the surgeon’s discretion.

All 53 CT scans obtained during the study period were reviewed by a fellowship-trained hand surgeon (JH) for presence of a PUC fragment, defined on the axial cut as a fracture that traversed the lunate facet in the coronal plane, exiting the palmar cortex between the lunate and scaphoid facets, and exiting ulnarly between the palmar and dorsal margins of the sigmoid notch. To control for technique consistency, measurements were made by one of the authors (JH), using 3-D MPR in Horos (Horos Project, Annapolis, MD, USA) (Zimmer et al., 2020). Horos allows for free manipulation of the three cardinal planes in a 3-D dataset, making it possible to obtain orthogonal cross-sectional images of fracture fragments displaced out of the axial plane of the index scan.

**Figure 1.**
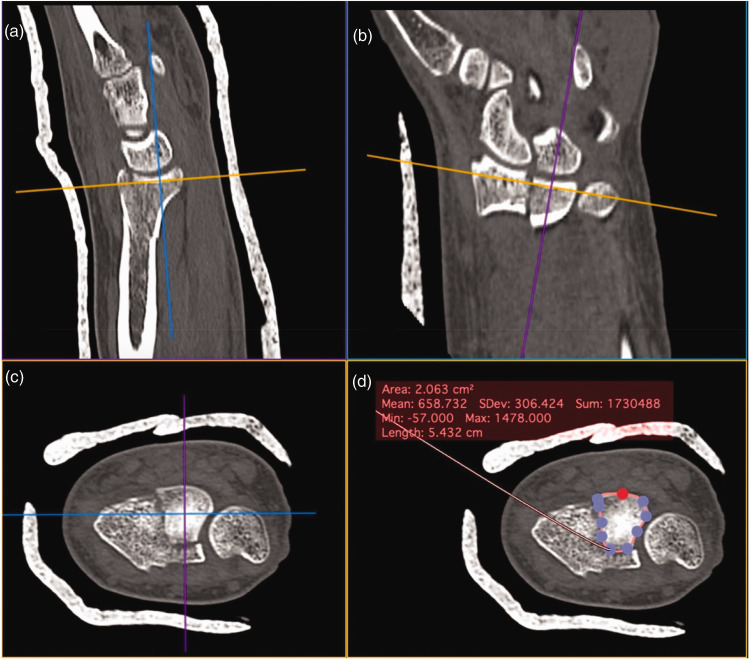
Measurement of the articular CSA of the PUC fracture fragment: (a) sagittal plane; (b) coronal plane. (c) The PUC fragment on the axial reconstruction and (d) Measurement of the CSA of the PUC fragment with closed polygon tool. CSA: cross-sectional surface area; PUC: palmar ulnar corner.

The closed polygon tool was used to measure the articular cross-sectional surface area (CSA) of the PUC fragment by aligning the tangent of the PUC fragment articular surface in the sagittal and coronal reconstructions. The area contained by the closed polygon was deemed the PUC fragment CSA (Figure 1). This was repeated for the remaining articular fragments to calculate the total distal radius articular surface area. The PUC fragment CSA was normalized as a percentage of the total distal radius articular surface area.

**Figure 2.**
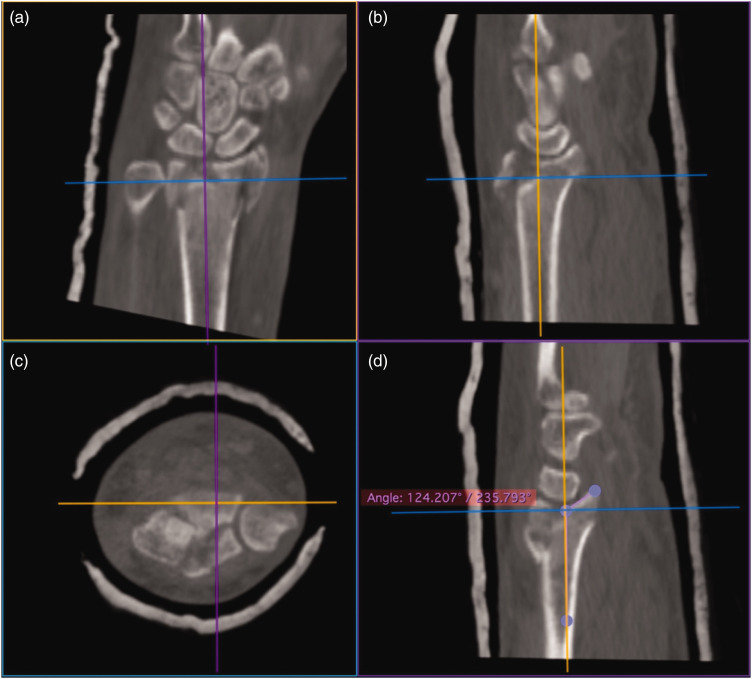
Measurement of the PUC fragment sagittal plane angulation entailed radial long axis alignment (middle of the articular surface through the middle of the diaphysis) on (a) coronal and (b) sagittal reconstructions. (c) PUC fragment surface on axial reconstructions and (d) The angle tool is being used to determine the angle between the long axis of the radius and the articular surface tangent of the PUC fragment. Purple line: sagittal plane of the radius. Yellow line: coronal plane of the radius. Blue line: transverse plane of the radius. PUC: palmar ulnar corner.

Sagittal plane angulation of the PUC fragment was measured by finding the radius long axis (midpoint of the metadiaphyseal canal on sagittal and coronal reconstructions), determining a line tangent to the PUC fragment articular surface in the sagittal plane, and using the angle tool to measure the angle between the long axis of the radius and the line tangent to the PUC fragment articular surface minus 90° (Figure 2). Positive values represent dorsal angulation relative to a line perpendicular to the long axis of the radius.

**Figure 3.**
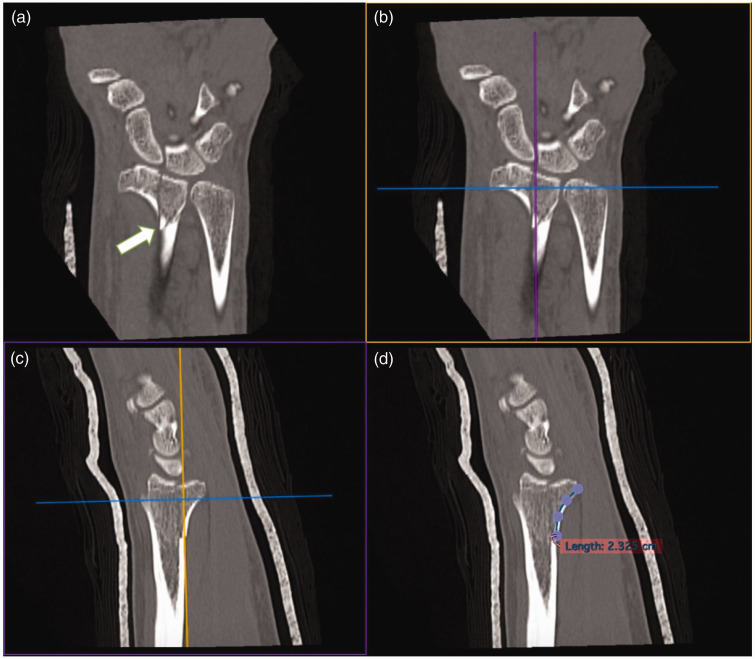
Measuring the maximum length of the palmar cortex of the PUC fragment. (a) Maximum proximal-distal length of the PUC fragment (white arrow) localized on coronal reconstruction tangential to palmar cortex. (b) Sagittal plane alignment with maximum proximal-distal length of the PUC fragment on coronal reconstruction. (c) Sagittal reconstruction with maximum proximal-distal length of the PUC fragment and (d) Open polygon tool being used to measure length of the PUC fragment palmar cortex from the proximal extent of the fracture to the most palmarly prominent point of the distal rim. PUC: palmar ulnar corner.

The maximum length of the palmar cortex attached to the PUC fragment was measured by finding the most proximal extent of the PUC fragment on the coronal reconstruction and measuring the corresponding palmar cortex length on the sagittal reconstruction. We used the open polygon tool to measure a curved line from the proximal aspect of the fracture to the most prominent volar point at the distal rim (Figure 3) (Beck et al., 2014).

**Figure 4.**
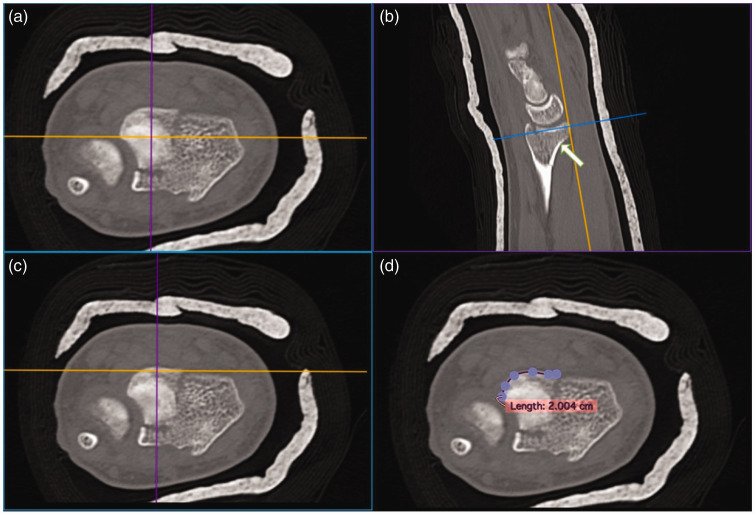
Measurement of the PUC fragment width at the palmar rim. (a) The PUC fragment localized in the axial plane. (b, c) The most palmarly prominent aspect of the articular rim was identified on axial and sagittal reconstructions at the intersection of the (b) orange and blue or (c) orange and purple lines, respectively and (d) The PUC fragment palmar rim and total distal radius palmar rim width were measured using the open polygon tool. This process was repeated to measure widest PUC fragment palmar cortical width proximal to the palmar rim (white arrow in b). PUC: palmar ulnar corner.

The radioulnar width of the PUC fragment was measured in two places: first, at the palmar rim; and second, at the widest part of the fragment proximal to the palmar rim. We defined the palmar rim as the transverse line across the distal radius, coinciding with the coronal plane tangent to the most palmar surface of the distal radius. A common term for this landmark is the watershed line, which refers to the bony landmark associated with the distal extent of the pronator quadratus (Benis et al., 2020). We defined this landmark as above for accuracy and consistency on CT imaging. The PUC fragment was localized on axial reconstructions and the PUC fragment palmar rim was identified on the sagittal reconstructions. On axial cuts, the open polygon tool was used to measure the radioulnar width of the PUC fragment corresponding to the palmar rim. Similarly, we measured the widest portion of the PUC fragment palmar cortex proximal to the palmar rim by scrolling incrementally through the PUC fragment axial cuts and measuring the widest portion of the PUC fragment proximal to the palmar rim (Figure 4). The width measurements of the PUC fragment at the palmar rim and the widest part of the PUC fragment proximal to the palmar rim were normalized as a percentage of the width of the entire palmar rim and of the entire metaphyseal width where the PUC fragment was the widest, respectively.

**Figure 5.**
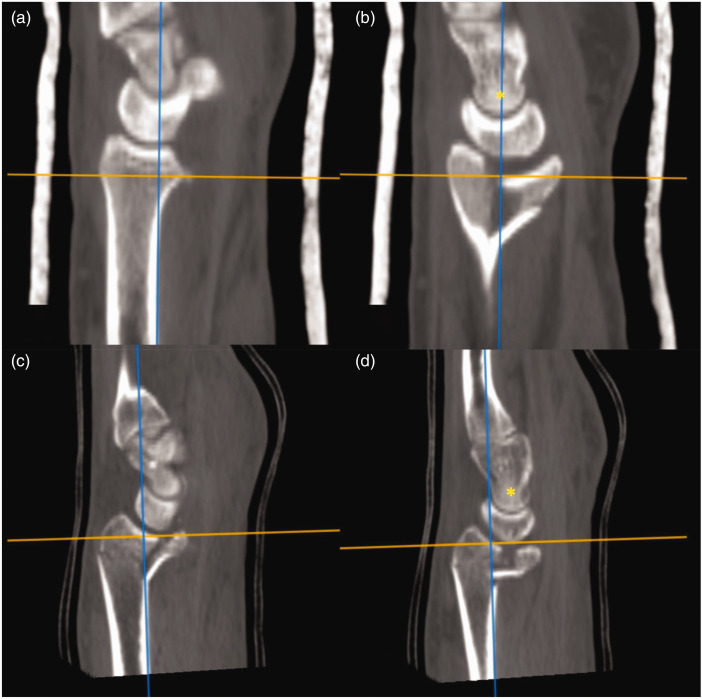
Determination of palmar radiocarpal translation in two different wrists. (a, c) Sagittal plane reconstructions showing anterior radial shaft palmar cortex. (b, d) Sagittal reconstructions scrolled until the capitate head centroid (yellow asterisk) is identified. (b) Normally the capitate head centroid aligns with the radial shaft volar cortex. In this fracture, radiocarpal subluxation is not present and (d) Demonstration of palmar radiocarpal subluxation. Blue vertical line: palmar cortex of the radius in coronal and sagittal planes. Yellow transverse line: perpendicular to palmar radial cortex representing the plane corresponding to the purely palmar translation component of capitate centroid displacement.

Palmar radiocarpal subluxation was assessed by aligning the palmar diaphyseal cortex of the radius on the coronal and sagittal planes. The centroid of the capitate head was found by scrolling through the sagittal reconstructions. Palmar radiocarpal subluxation was deemed present if the centroid of the capitate head was palmar to the line drawn tangential to the palmar cortex of the radius in the sagittal plane (Figure 5) (Kuhnel et al., 2019).

**Figure 6.**
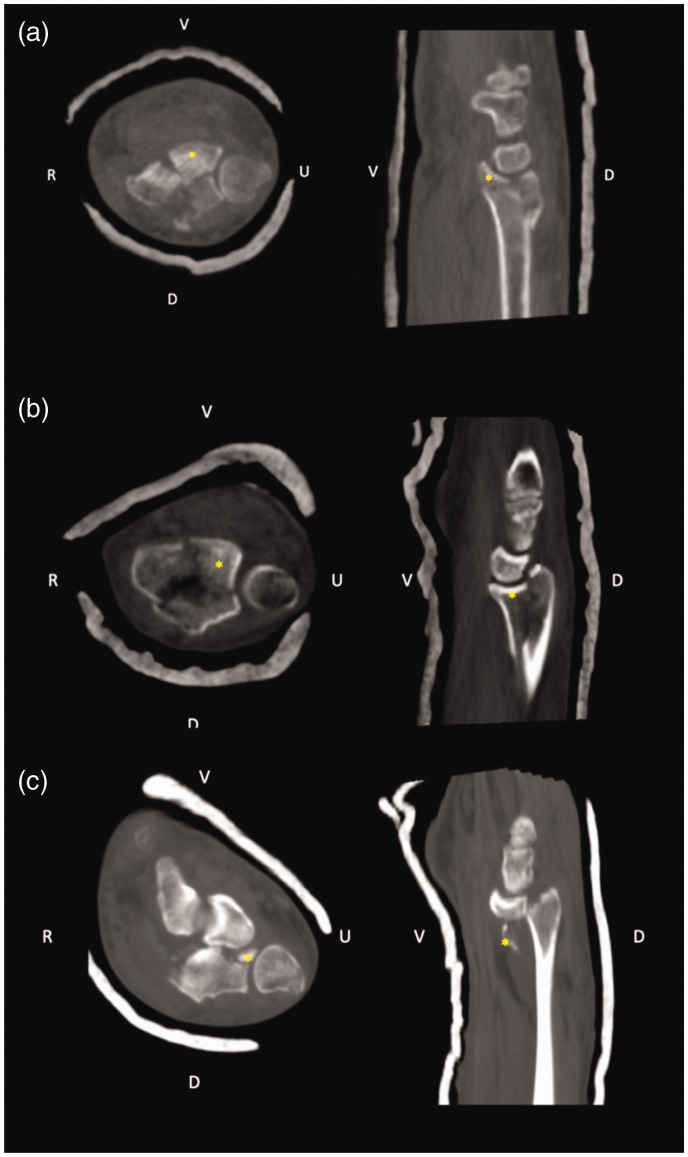
Computed tomography reconstructions demonstrating the axial and sagittal profiles of the (a) type 1 dorsally extended fragment, (b) type 2 large palmar shear fragment and (c) type 3 small palmar rim fragment. Asterisk denotes the palmar ulnar corner fragment.

**Figure 7.**
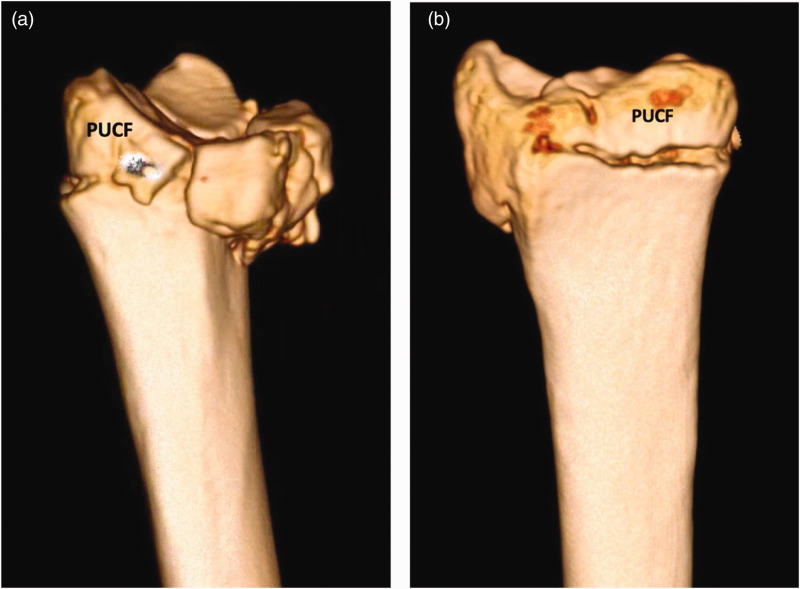
(a) 3-D reconstruction of a distal radial fracture with a type 1 palmar ulnar corner fragment from the ulnar and (b) from the anterior perspective.

**Figure 8.**
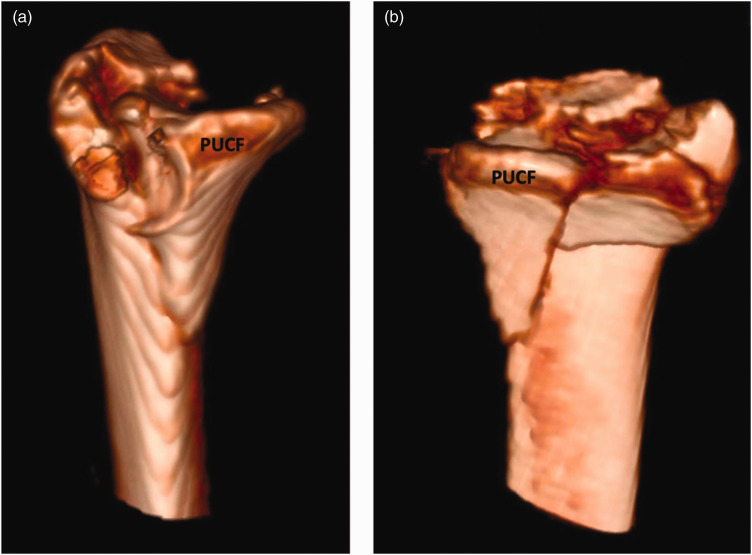
(a) 3-D reconstruction of a distal radial fracture with a type 2 palmar ulnar corner fragment from the ulnar and (b) from the anterior perspective.

**Figure 9.**
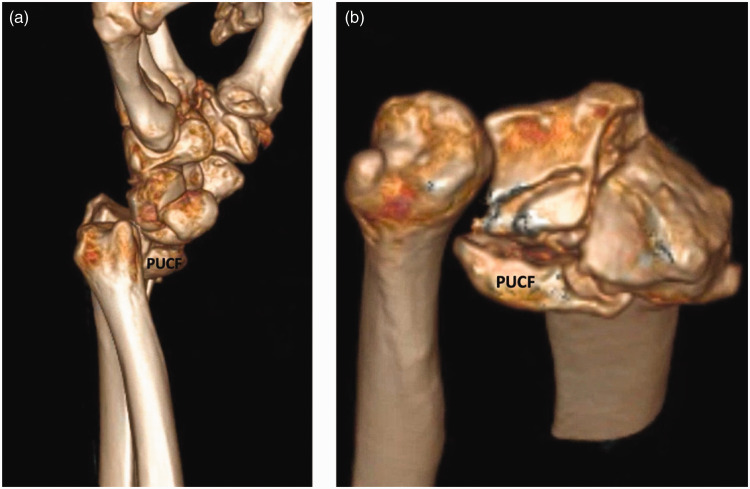
(a) 3-D reconstruction of a distal radial fracture with a type 3 palmar ulnar corner fragment from the ulnar perspective, with carpus included showing radiocarpal subluxation, and (b) from the articular surface perspective.

Three morphological types of PUC fragments emerged, based on 3-D CT MPR measurements (Figure 6). Type 1 fragments were defined as dorsally rotated in the sagittal plane (Figures 6(a) and 7). Type 2 fragments had a large palmar cortex with a vertical fracture line consistent with a shear mechanism (Figures 6(b) and 8). Type 3 fragments were relatively small avulsion or comminuted palmar rim fragments (Figures 6(c) and 9).

### Statistical analysis

To determine the distribution of PUC fragment type in our population, the frequency of each PUC fragment type was calculated. Two of the authors (AC, CC) classified PUC fragments for 33 cases and an interrater correlation coefficient (ICC 2,1) was calculated to determine the inter-observer reliability. For each measurement, data were screened for normality using the Shapiro–Wilk test to support the use of parametric statistics. To determine if different PUC fragment types had different morphology and displacement characteristics, the means for each measurement were compared between the three groups using one-way ANOVA with Tukey’s test for multiple comparisons. The proportion of fragments associated with palmar radiocarpal subluxation was calculated, and comparisons of proportions between PUC fragment types were performed using Fisher’s exact test. Statistical significance was set at *p* < 0.05. All data are reported as mean (SD).

## Results

In total, 53 patients with 53 intra-articular distal radial fractures and who had preoperative CT scans were identified. A total of 40 fractures had a PUC fragment. The others were excluded. Of the 40 PUC fragments, 12 were type 1, 21 were type 2 and seven were type 3 fragments. Agreement between raters yielded an ICC of 0.68, indicating moderate agreement between raters.

The mean articular CSA was 2.1 cm^2^ (SD 0.8) for type 1 fragments, 1.5 cm^2^ (SD 0.4) for type 2 fragments and 0.7 cm^2^ (SD 0.1) for type 3 fragments. The mean CSA of fragment type as a percentage of the distal radius articular surface was 37% (SD 9.5) for type 1 fragments, 28% (SD 10.0) for type 2 fragments and 13% (SD 2.2) for type 3 fragments. Mean PUC fragment articular CSA was significantly larger for type 1 fragments when compared with both type 2 (*p* = 0.03) and type 3 (*p* < 0.001) fragments and for type 2 fragments when compared to type 3 fragments (*p* = 0.03). These differences remained statistically significant after normalizing the PUC fragment articular CSA as a percentage of the total distal radius CSA.

The mean sagittal plane angulation of the PUC fragment was 36° (SD 15) for type 1 fragments, 11° (SD 15) for type 2 fragments and 10° (SD 14) for type 3 fragments. Type 1 fragments were significantly more rotated in the sagittal plane than type 2 (*p* < 0.001) and type 3 (*p* = 0.003). fragments. There was no significant difference in sagittal rotation between type 2 and type 3 fragments (*p* = 0.98).

The mean palmar cortex length of PUC fragments was 14.3 mm (SD 3.8) for type 1 fragments, 18.3 mm (SD 4.5) for type 2 fragments and 8.5 mm (SD 1.3) for type 3 fragments. Type 2 fragments had a significantly longer palmar cortex than type 1 (*p* = 0.02) and type 3 (*p* < 0.001) fragments, while type 1 fragments had a longer palmar cortex compared to type 3 fragments (*p* = 0.01).

The mean width at the palmar rim of PUC fragments was 20 mm (SD 4.2) for type 1 fragments, 19.3 mm (SD 4.3) for type 2 fragments and 14.6 mm (SD 8.3) for type 3 fragments. Type 1 fragments comprised 58% (SD 8.6), type 2 fragments 57% (SD 12) and type 3 fragments 41% (SD 16) of the width of the palmar rim. Fragment types did not significantly differ in absolute PUC fragment width (*p* = 0.08). In terms of percentage of the palmar rim width, type 3 fragments were smaller than both type 1 (*p* = 0.01) and type 2 (*p* = 0.01) fragments.

The mean maximal metaphyseal width proximal to the palmar rim of PUC fragments was 16.1 mm (SD 4.1) for type 1 fragments and 12.3 mm (SD 3.2) for type 2 fragments. Type 1 fragments comprised 56% (SD 10) and type 2 fragments 44% (SD 12) of the width of the palmar metaphysis. The type 3 fragment width at the palmar metaphysis proximal to the palmar rim could not be measured reliably due to being very distal. The absolute width and percentage of the palmar metaphyseal cortical width for type 2 fragments were smaller than type 1 fragments (*p* = 0.006).

Palmar radiocarpal subluxation was present in none of the type 1 fragments (*n* = 0/12), 12 of the type 2 fragments (*n* = 12/21) and all type 3 fragments (*n* = 7/7). A significant difference in the presence of subluxation between fragment type was found (*p* < 0.001).

## Discussion

We identified three types of PUC fragments in 40 wrists with intra-articular distal radius fractures. Type 1 fragments were dorsally angulated, had a large articular portion, intermediate palmar cortical length and width, and no instances of palmar radiocarpal subluxation. Type 2 fragments had an intermediate articular portion, a long palmar cortex, averaged over half palmar rim width, tapered proximally and were associated with palmar radiocarpal subluxation in over half of cases. Type 3 fragments had minimal articular surface and palmar cortical length, averaged less than half of palmar rim width and were associated with palmar radiocarpal subluxation in all cases. Type 2 fragments were most common.

In 23 association for osteosynthesis (AO) C3 DRFs, Li et al. (2020) found the mean ‘volar medial’ fragment CSA was 1.5 cm^2^ (SD 0.8), consistent with our study where the mean CSA for all fragment types was 1.6 cm^2^ (SD 0.7). After defining three PUC fragment types based on morphological data, each had significantly different articular CSAs.

Benis et al. (2020) studied 3-D CT scans in 61 DRFs with palmar lunate facet fragments. They were divided into three types: long palmar cortex; short palmar cortex; and fragments extending beyond the lunate facet into the scaphoid fossa. Fragments were categorized according to AO and Fernandez classifications. Fractures with a short palmar cortex did not correlate with any AO or Fernandez classification fracture type; however, there was a significant correlation with longer palmar cortical fragments classifying as AO B3 and Fernandez type II shearing fractures and shorter fragments classifying as AO C and Fernandez type III compression. There was no difference in fragment CSA between fracture types according to the AO and Fernandez classifications. It is impossible to correlate the Benis et al. (2020) classification with ours due to methodological differences in the studies. Further, the type 3 fragments from Benis et al. (2020) were not part of our study. However, both studies support the existence of different lunate facet fragment morphologies, each of which could behave differently, warranting consideration of alternative fixation strategies. Neither study investigated treatment (Benis et al., 2020).

Apergis et al. (2002) reported five cases of isolated PUC fragments treated non-operatively, all resulting in palmar carpal translation, highlighting the critical role of the PUC in maintaining radiocarpal stability. Surgical repair was recommended.

Anterior locking plates may not capture all PUC fragments. Harness et al. (2004) reported seven patients who lost PUC fragment reduction after anterior plate fixation, resulting in limited wrist motion, loss of grip strength, deformity and pain. While the palmar surface of the distal radius is mostly flat, the distal 5 mm of the PUC projects 3 mm (SD 1) palmar to the palmar radial cortex (Andermahr et al., 2006). Strategies to capture this fragment with anterior locking plates include a distal ulnar extension or distal plate placement, which is concerning for flexor tendon rupture (Kachooei et al., 2016; Kitay et al., 2013; Soong et al., 2011).

Numerous fixation strategies have been devised for the PUC fragment (Bakker and Shin, 2014; Beck et al., 2014; Chin and Jupiter, 1999; Guerrero et al., 2020; Moore and Dennison, 2014; O’Shaughnessy et al., 2015; Ruch et al., 2004; Waters et al., 2014; Weisler et al., 2006). Beck et al. (2014) reviewed 52 AO B3 DRFs treated with an anterior locking plate with a distal ulnar extension to capture small PUC fragments, and still had reduction failure in 7 (14%) patients with significantly worse motion than when reduction was maintained. The only risk factor for PUC fragment fixation failure was a palmar cortex length less than 15 mm. In our study, type 1 and 3 fragments had mean palmar cortex lengths less than 15 mm and accounted for 47% of our PUC fragments. This implies that many of our patients were at risk for reduction loss with anterior plate fixation.

Successful treatment may depend on recognizing fragment type (Beck et al., 2014; Harness et al., 2004). In our study, type 1 fragments were dorsally malrotated in the sagittal plane, necessitating derotation to restore palmar tilt. Type 2 PUC fragments were large with a long palmar cortex, which we believe can be fixed with a buttress technique. Type 3 small palmar rim or comminuted fragments are the most troublesome and were all associated with palmar radiocarpal subluxation. The type 3 palmar cortex is so small that it may not be captured by an anterior locking plate (Beck et al., 2014; Harness et al., 2004). Based on our data, type 3 fragments do not cross the radioulnar mid-axis. O’Shaughnessey et al. (2016) suggested that the PUC fragment must be at least 7 mm wide from radial to ulnar, 4 mm wide from palmar to dorsal and 5 mm long from proximal to distal to accept an anterior hook plate (TriMed, Valencia, CA, USA). While there is some overlap of these dimensions with our measurements, some type 3 fragments were smaller, indicating alternative fixation methods (Moore and Dennison, 2014). When stable fixation cannot be achieved, bridge plating may be a strategy to dorsally translate the carpus on the distal radius, resisting forces acting to palmarly displace the radiocarpal joint and the PUC fragment (Guerrero et al., 2020). PUC fragments did not displace >1 mm with loads up to 300 N in a biomechanical study of intra-articular DRFs treated with a dorsal bridge plate (Guerrero et al., 2020).

Our study has several weaknesses. We used Kuhnel’s (2019) technique to determine radiocarpal subluxation, which they defined using radiographs and may not be applicable to CT scans. Radiocarpal alignment may be influenced by wrist position, which was not standardized for our CT scans. However, we still believe our methodology is valid. Another weakness was only moderate reliability of two authors agreeing which type was which when viewing 33 CT case examples. This was despite our defining the three fragment types based on morphological parameters. Another limitation is that we did not address the role articular fragments other than the PUC fragment may play in fracture stability and fixation choices.

We identified three fragment types in our cohort and acknowledge there could be others. We also acknowledge the ability to classify PUC fragments is imperfect. Nevertheless, each type may warrant different treatment tactics, and further studies are needed to determine the optimal fixation for each fragment type.
